# An Awful Blunder

**Published:** 1890-06

**Authors:** G. W. Emery

**Affiliations:** Minneapolis


### AN AWFUL BLUNDER.

DR. G. W. EMERY, MINNEAPOLIS.

In the month of December last, the writer, while in a neighboring State, on business, and a guest of a prominent citizen and kindred, unearthed the following terrible, unique, and yet interesting case such as few meet with in a life-long practice, and of which several of our leading authorities make no mention.

The writer had finished his MS. with a query as to how it might be received by his professional compeers when his attention was directed, by Dr. A. A. Pine, of St. Paul, to a very similar case recorded by Prof. Byford in his obstetrical work, pages 454–55. The awful possibility of the recurrence of the event leads me to hope that this may be of interest and benefit to your many readers.

On the first day of October, 1889, the wife of my host, a young multipara aged about twenty-four years, strong, robust, in fact, the very picture of health, was taken in her second labor (her first a twin pregnancy, children eight and ten pounds respectively, one still-born, the other living two or three weeks).

In the labor to be described, primary pains commenced about four o'clock P. M. on the date above; patient retired to her chamber and to bed. About two hours afterward a messenger was dispatched for Dr. T., her former attendant, and a physician of forty years' practice in the town. Labor appeared to be perfectly normal, and at ten o'clock P. M. a healthy female child was born, no anæsthetic was used, nor did the patient act as though the labor had been of more than ordinary severity. After the birth the usual pleasantries were passed, and for about half an hour patient seemed to rest easy and normally, making pleasant remarks to the friends and physician. The husband, feeling all danger past, retired to his study to hastily write a few business letters; he had left the chamber but a few minutes when the doctor remarked, "I must now get the after-birth," and proceeded to manipulate for the same. The patient soon began to give expressions to feelings of pain, and the doctor (for the first time in the case) instructed one of the ladies present, or the nurse, to administer Chloroform.

With the administration of the anæsthetic increased effort and force were used on the part of the physician, and the husband was summoned and placed in charge of the administration of the anæsthetic, while the doctor seemed to increase his power and energy to dislodge (as he claimed) the after-birth, with hand and forearm inserted into the vagina and pelvic cavity or uterus, and perspiration dropping from his face, so that the onlookers frequently wiped his face with towels, while the patient's expressions of agony were so intense as to be almost unendurable to those present. The doctor, to the several inquiries as to "What is the matter?" would respond, "The after-birth is attached to every part of the womb." With the patient evidently growing weaker and entering the stage of collapse, respiration increasing rapidly and face assuming an ashy pallor; the husband noticed the radial pulse was gone and the hands and extremities cold, and thinking she was dead, stepped to an adjoining window and broke into tears and sobbings, to be sharply reprimanded by the physician with the exclamation, "Why, what's the matter with you? You act as though Emma was dying; she's all right, give the Chloroform."

The nurse states that at this time she was satisfied the patient was dying, and ventured the suggestion that the doctor had better send for counsel, to be informed with considerable asperity and tartness that "he (the doctor) knew his business, and no necessity for assistance existed."

Thus, from half-past ten till about twelve o'clock, was this terrible forced manipulation progressing, till suddenly Dr. T. exclaimed, "Send as quickly as possibly for Dr. R.," who, responding to the hurried messenger, arrived at half-past twelve, and immediately proceeded to examination, and inserting hand and forearm, quickly, and amidst the greatest excitement on his part, disclosed from the vulva a large, round body, and exclaimed, "Inversion." Then, after examining it carefully, turning it over from hand to hand, and with the greatest agitation, he in a subdued voice exclaimed, "Dr. T., will I cut this off?" or, "Hadn't I better cut this off?" when Dr. T. responded, "Yes, I guess so," and, as Dr. R. was slightly deaf, the husband, leaning over to Dr. T., remarked, "What is he going to cut off, Doctor? See that he don't do any harm." Dr. T. responded, "It's all right if he don't cut too high," and turning, remarked in a loud tone of voice to Dr. R., "Be careful, Doctor!" Dr. R., apparently greatly agitated, then called for a pair of scissors and a piece of string. The scissors not being found, he asked for a knife, and the husband handed him his penknife and the membrane was cut and the object removed. Dr. R. almost immediately retired, taking with him the removed mass, and in a few minutes the patient was dead.

The above detailed account is entirely corroborated by all present. But great dissatisfaction was expressed at the methods and forcible manipulations of Dr. T., and confusion as to whether Dr. R. was not in part responsible for the death by his cutting operation, though it was conceded that they (the friends) thought the patient about dead when Dr. R. arrived.

With a desire to remove these feelings, the writer decided on an interview with the physician in attendance. A call upon Dr. T. resulted in the doctor stating that "It was very sad, but it was the most terrible case of attached placenta that he had ever met." To an inquiry as to whether inversion existed, he stated: "No; Dr. R. thought so at first, but it was a mistake." To the query as to whether the womb had been removed, the doctor positively entered an emphatic denial, exclaiming, "Do you think I'm a fool? Haven't I had experience enough to keep me from doing such an act?" The writer stated he thought he ought to have, and suggested that the friends were urging the exhuming of, and a post-mortem on, the body, and if it were done, it was their wish that he be present. This caused an intense expression of anxiety and great agitation to the doctor, and to the inquiry as to what Dr. R. had removed, he, Dr. T., promptly responded, "Why, the after-birth." After some further conversation, the writer, informing him that he should immediately proceed to Dr. R.'s office and examine the placenta, left, and in a few minutes was in Dr. R.'s office, and proceeded to state that he called to see the after-birth of Mrs. E. M., which Dr. T. informed him he, Dr. R., had taken away and was in his possession. Dr. R. denied having an after-birth, and the writer demanded permission to see what he had removed. Dr. R. quite excitedly proceeded to express the horrors of the case, and greatly regretted the necessity of complying with the request, asking earnestly for as much leniency as possible for the attending physician, and then Dr. R. produced the womb. Recounting the facts as above stated, he proceeded to say that he had arrived at the bedside of Mrs. M. at half-past twelve at night, noticed the collapsed condition and deathly expression of countenance, and immediately proceeded to examination, when his hand passed into the intestinal cavity and came in contact with the convoluted folds of intestines. Extracting his hand, it was followed by the uterus, held by a small portion of the vaginal tissue at the cul-de-sac. He, Dr. R., said he was utterly lost as to comprehending its condition, and suggested inversion, but on closer examination found it to be the womb, enucleated except at the point indicated, and the patient dying. He, Dr. R., removed as above stated and retired, knowing full well the result but not waiting to witness it. He, Dr. R., said he had been passing through a terrible mental struggle as to his duty. He had, on the second day after the death of the mother, taken the organ to a leading physician (Dr. B.) of a neighboring city, shown it to him, and asked his advice, who informed Dr. R. that, as the patient was dead, and Dr. R. and Dr. T. being old practitioners in the same field, he, Dr. R., had better say nothing about the affair, but keep the organ carefully. He, Dr. B., further stated he had never seen such a terrible piece of work in his life before. This advice on the part of Dr. B., Dr. R. informed me, had kept him quiet, though his wife had urged him to explain the facts to the husband. To the inquiry as to where the placenta was, he, Dr. R., responded that he had never seen it; he said that in proceeding to the examination he groped about the labies externally to find the cord, but it was not there. He then went internal, but did not find it nor any other indications of the cord or placenta. The organ produced measured eleven (11) inches in length and seven and one-half ($7\frac{1}{2}$) inches in diameter through centre of body, one ovary and ligaments much larger than the other. Mouth patulous and ragged, organ rent on left side up to ligamental attachments. Internal surface denuded of decidual membrane and the muscular tissue torn up and into, as though gnawed by an animal; this was by the finger of Dr. T. The point at which the cutting by Dr. R. was made was very apparent, and agreed with his (Dr. R.'s) statement. And with the writer, he, Dr. R., agreed that the after-birth had slipped through the rent and was then in the body of the deceased.

Another interview was had with Dr. T., and the facts (of which he had been fully aware, because the organ was exhibited to him two days after the death, by Dr. R.) he, Dr. T., now stoutly denied any force on his part in the treatment of the case, and in the most contemptuous manner, by an implication, proceeded to throw the blame on Dr. R. He further attempted to claim that the decidual membrane, so horribly mutilated and denuded from the internal surface, was the placenta, but finally abandoned this position and conceded the probability of the loss of the placenta in the intestinal cavity, after which concession the husband thought a post-mortem unnecessary. The age, respectability, and former standing of this horrible blunderer, moved feelings of pity on the part of those who were so awfully injured, though a liberal amount is demanded for the use of the motherless babe.

In conclusion, the writer would suggest for the consideration of your readers the following interrogatories which have very forcibly come to his mind, viz.:

First. Was the advice given by Dr. B. to Dr. R. proper or justifiable under the circumstances?

Second. Was the action of Dr. R. in attempting to follow the advice commendable or censurable?

Third. Was the action of the friends in being willing to condone the death and thus thwart demands of civil law, thus permitting this Dr. T. to continue in active practice, right or wrong?

The writer would be glad to have these questions answered. —*Northwestern Medical Journal.*

